# Influenza A H1N1 Infection Triggers Endothelial Immunothrombosis via TNF-α–Tissue Factor–Thrombin Signaling in Human Brain Microvascular Endothelial Cells

**DOI:** 10.3390/cimb48101043

**Published:** 2026-10-09

**Authors:** Luis A. Pérez-Moreno, Silvia Guzmán-Beltrán, Víctor A. Hernández-Hernández, Blanca H. Ruiz-Ordaz, Belen Romo-Espinosa, Carlos Cabello-Gutiérrez

**Affiliations:** 1 Departamento de Investigación en Virología y Micología, Instituto Nacional de Enfermedades Respiratorias “Ismael Cosio Villegas”, Ciudad de México 14080, Mexico; luis_perez_86@hotmail.com (L.A.P.-M.); viherher@mail.unitec.mx (V.A.H.-H.); belenromoespinosa@gmail.com (B.R.-E.); 2 Posgrado en Ciencias Biológicas, Universidad Nacional Autónoma de México, Ciudad de México 04510, Mexico; 3 Departamento de Investigación en Microbiología, Instituto Nacional de Enfermedades Respiratorias “Ismael Cosio Villegas”, Ciudad de México 14080, Mexico; sguzman@iner.gob.mx; 4 Laboratorio de Simulación, Universidad Tecnológica de México, Ciudad de México 11320, Mexico; 5 Departamento de Biología Molecular y Biotecnología, Instituto de Investigaciones Biomédicas, Universidad Nacional Autónoma de México, Ciudad de México 04510, Mexico; bhro@iibiomedicas.unam.mx

**Keywords:** influenza A virus, H1N1, endothelial cells, tissue factor, TNF-α, thrombin, immunothrombosis, endothelial dysfunction, coagulation, inflammation

## Abstract

Severe Influenza A virus (IAV) infection is associated with endothelial dysfunction, inflammation, and coagulation abnormalities. However, the mechanisms linking endothelial inflammatory activation to thrombosis remain incompletely understood. We investigated whether Influenza A/H1N1 induces an immunothrombotic phenotype in human brain microvascular endothelial cells. We infected HBEC-5i cells with Influenza A/H1N1 pdm09. We assessed viral infection by RT-qPCR detection of matrix (M) transcripts and by immunoblotting for the M1 protein. We evaluated endothelial responses by measuring TNF-α expression and secretion, Tissue Factor (TF) expression, TF-dependent factor Xa generation, and responses to recombinant TNF-α and thrombin. H1N1 infection increased TNF-α expression and secretion and enhanced TF expression and functional activity, indicating acquisition of a procoagulant endothelial phenotype. Recombinant TNF-α reproduced infection-associated TF induction, whereas thrombin enhanced TNF-α production and TF activity, supporting reciprocal amplification between inflammatory and coagulation pathways. These findings support a TNF-α–TF–thrombin feed-forward signaling circuit linking endothelial inflammation and coagulation during H1N1 infection. Influenza A/H1N1 induces an immunothrombotic phenotype in human brain microvascular endothelial cells, identifying this signaling axis as a potential mechanistic target for influenza-associated vascular and thrombotic complications.

## 1. Introduction

Influenza A virus (IAV) remains a major global public health threat, causing seasonal epidemics and sporadic pandemics that result in substantial morbidity and mortality [1]. Although influenza has traditionally been regarded as a respiratory disease, growing clinical and experimental evidence indicates that severe infection extends far beyond the lungs, affecting the vascular system and contributing to systemic complications, including endothelial dysfunction, microvascular thrombosis, disseminated intravascular coagulation, and multiorgan failure [2,3]. These vascular manifestations are increasingly recognized as critical determinants of disease severity and poor clinical outcomes. Consequently, the vascular endothelium has emerged as an active participant in influenza pathogenesis rather than a passive barrier, integrating inflammatory and hemostatic responses that ultimately determine the magnitude of tissue injury and host outcome [4].

The vascular endothelium is a dynamic immunoregulatory organ that maintains vascular homeostasis by regulating permeability, leukocyte trafficking, coagulation, fibrinolysis, and inflammatory signaling. Under physiological conditions, endothelial cells maintain a tightly regulated anticoagulant phenotype through the coordinated activity of thrombomodulin, endothelial protein C receptor, tissue factor pathway inhibitor, and other protective mediators [5]. During viral infections, however, inflammatory stimuli alter endothelial function, promoting cytokine production, leukocyte adhesion, disruption of intercellular junctions, and a procoagulant phenotype. This transformation is a hallmark of severe viral infections [2,6].

Among the molecular pathways governing endothelial immunothrombosis, Tissue Factor (TF), a 47-kDa transmembrane glycoprotein, is the principal initiator of the extrinsic coagulation pathway and a key regulator of inflammatory signaling [7]. Upon binding activated factor VII (FVIIa), TF activates factor X (FX), promotes thrombin formation and fibrin deposition, and modulates protease-activated receptors (PARs), particularly PAR-1 and PAR-2, thereby amplifying Nuclear factor kappa-light-chain-enhancer of activated B cells (NF-κB)-dependent inflammatory responses and cytokine production [6,8]. Tumor necrosis factor-alpha (TNF-α), a central mediator of influenza-associated inflammation, potently induces endothelial TF expression and is strongly associated with disease severity, vascular leakage, and thrombotic complications [3,9]. In turn, thrombin functions not only as the terminal effector of coagulation but also as a potent inflammatory mediator that reinforces endothelial activation through PAR-1 signaling, suggesting a self-amplifying inflammatory–coagulation circuit. Although these interactions have been extensively investigated in sepsis and, more recently, in COVID-19-associated immunothrombosis, considerably less is known about how IAV directly modulates these pathways in human brain microvascular endothelial cells [3,10,11].

The close interplay between inflammation and coagulation has led to the concept of immunothrombosis, a host defense mechanism in which coagulation activation helps control pathogens and amplifies innate immune responses. Although initially protective, excessive immunothrombosis can cause endothelial dysfunction, microvascular thrombosis, impaired tissue perfusion, and organ failure [12,13]. Although immunothrombotic mechanisms have been extensively studied in SARS-CoV-2 infection, evidence suggests that similar pathways are activated during severe influenza [10]. However, the cellular mechanisms that trigger endothelial immunothrombosis in influenza remain poorly understood.

Brain endothelial cells form the structural and functional core of the blood–brain barrier and are essential for regulating neurovascular homeostasis [14]. Growing evidence indicates that endothelial activation contributes to the neurological manifestations of severe influenza, including encephalopathy, cerebrovascular complications, and blood–brain barrier disruption [4,11]. However, whether Influenza A(H1N1) pdm09 directly reprograms brain endothelial cells toward an endothelial immunothrombotic phenotype through coordinated activation of inflammatory and coagulation signaling remains unknown. We therefore hypothesized that productive Influenza A(H1N1) pdm09 infection promotes endothelial immunothrombosis by establishing a TNF-α–TF–thrombin signaling circuit that perpetuates inflammatory and procoagulant activation. To test this hypothesis, we investigated viral replication, TNF-α expression, TF induction and functional activity, and the reciprocal interactions between TNF-α and thrombin signaling in human brain microvascular endothelial cells.

## 2. Materials and Methods

### 2.1. Cell Culture

Human brain microvascular endothelial cells (HBEC-5i from the American Type Culture Collection (ATCC, Manassas, VA, USA)) were maintained in Dulbecco’s Modified Eagle Medium/Nutrient Mixture F-12 (DMEM/F12; Gibco, Thermo Fisher Scientific, Waltham, MA, USA) supplemented with 10% heat-inactivated fetal bovine serum (FBS; HyClone, Cytiva, Logan, UT, USA) and 40 μg/mL endothelial cell growth supplement (ECGS; Sigma-Aldrich, Merck, St. Louis, MO, USA). We cultured cells at 37 °C in a humidified atmosphere with 5% CO_2_ and passaged them at 80–90% confluence using standard cell culture procedures. We used only subconfluent cultures with viability >95%, as determined by trypan blue exclusion, in all experiments.

We cultured Madin-Darby canine kidney cells (MDCK; ATCC, Manassas, VA, USA) in Eagle’s Minimum Essential Medium (EMEM; ATCC, Manassas, VA, USA) supplemented with 10% heat-inactivated FBS and maintained them at 37 °C in 5% CO_2_. We routinely used MDCK cells for Influenza A virus propagation and plaque titration.

For all experiments, we seeded HBEC-5i cells in tissue culture plates at assay-specific densities and allowed them to reach approximately 80% confluence before infection or stimulation. We replaced the cell culture medium with fresh complete medium 24 h before each experiment to minimize variability from nutrient depletion or soluble factor accumulation. We routinely monitored all cell cultures for morphology and periodically tested them to confirm the absence of mycoplasma contamination. This standardized culture protocol ensured reproducibility across biological replicates and experimental conditions.

### 2.2. Virus Propagation and Titration

Influenza A virus (H1N1) strain A/California/07/2009 (H1N1) pdm09 (ATCC^®^ VR-1894^TM^, Manassas, VA, USA) was used in this study. Viral matrix (M) gene sequences were referenced against the Influenza A virus (H1N1) strain A/California/07/2009 (H1N1) pdm09 segment 7 sequence deposited in NCBI (Ref Seq accession no. NC_026431.1). We propagated virus stocks in confluent MDCK cells infected at a multiplicity of infection (MOI) of 0.1. We incubated infected cultures at 37 °C in a humidified atmosphere with 5% CO_2_ until cytopathic effects exceeded 90% (approximately 72 h post-infection). We collected culture supernatants, clarified them by centrifugation at 900× *g* for 10 min to remove cellular debris, then aliquoted and stored them at −80 °C until use.

We determined viral titers by hemagglutination assay using type O-positive human erythrocytes. Briefly, we prepared two-fold serial dilutions of viral supernatants in phosphate-buffered saline (PBS) in 96-well plates and added an equal volume of 0.5% erythrocyte suspension. After incubation at 37 °C for 1 h, we assessed hemagglutination visually. We recorded the highest viral dilution showing complete hemagglutination as the hemagglutination titer. We confirmed viral titers by lytic blade tests.

Before each experiment, we quantified viral infectivity using standard plaque assays on confluent MDCK monolayers. We calculated and reported viral titers in plaque-forming units per milliliter (PFU/mL).

Before each experiment, we measured Influenza A virus infectivity by plaque assay on confluent MDCK cell monolayers. We seeded MDCK cells into 24-well plates and incubated them until they reached 90–100% confluence. We prepared serial tenfold dilutions of each virus stock in serum-free infection medium.

We washed cell monolayers once with PBS and inoculated them with 200 μL of each viral dilution. After allowing the virus to adsorb for 1 h at 37 °C in a humidified atmosphere with 5% CO_2_, we removed the inoculum after gently rocking every 15 min to promote even distribution. We then overlaid the cells with an infection medium containing 1.2% methylcellulose and TPCK-treated trypsin (2 μg/mL), which limits viral spread and facilitates localized plaque formation.

We incubated the infected cultures at 37 °C in 5% CO_2_ for 96 h. After incubation, we gently removed the overlay medium, washed the cell monolayers with PBS, and fixed them with 4% paraformaldehyde for 20 min at room temperature. We then stained the cells with 0.1% crystal violet in 20% methanol for 15–20 min, rinsed thoroughly with distilled water, and air-dried. We manually counted plaques in wells containing 10–100 discrete plaques. Viral titers were calculated from wells containing countable plaques and expressed as plaque-forming units per milliliter (PFU/mL), according to the following equation:

**PFU/mL** = (Number of plaques × Dilution factor)/Volume of inoculum (mL)

We titrated virus stocks independently before each experiment and performed all plaque assays in duplicate. We reported viral infectivity as PFU/mL and used it to normalize the MOI in subsequent experiments.

### 2.3. Viral Infection

We seeded HBEC-5i cells at the appropriate density for each assay and allowed them to reach approximately 80% confluence before infection. We infected cells with Influenza A virus (H1N1) pdm09 at an MOI of 5. Virus adsorption was performed for 1 h at 37 °C with gentle rocking to ensure homogeneous infection. After adsorption, we removed the inoculum, washed cells with PBS to remove unbound viral particles, and added fresh culture medium. We treated uninfected cells identically and used them as experimental controls. We collected cells and culture supernatants at the indicated time points for subsequent molecular, biochemical, and functional analyses. We selected an MOI of 5 based on preliminary optimization experiments and previous reports demonstrating efficient endothelial infection while preserving cell viability.

### 2.4. Cell Viability Assay

We seeded HBEC-5i cells in 6-well plates at 1.8 × 10^6^ cells per well and maintained them under serum-free conditions. Cells were either left uninfected or infected with Influenza A virus (H1N1) pdm09 at MOI = 5 and incubated for 48 h at 37 °C in a humidified atmosphere containing 5% CO_2_. At the end of the incubation period, cells were collected, and cell viability was determined by the trypan blue exclusion assay using 0.4% trypan blue solution. Viable and non-viable cells were counted using a Neubauer hemocytometer. Cell viability was expressed as the percentage of viable cells relative to the total number of cells counted. Three independent experiments were performed.

### 2.5. Recombinant TNF-α and Thrombin Stimulation

To investigate the contributions of inflammatory and coagulation signaling pathways to endothelial activation, HBEC-5i cells were stimulated with recombinant human TNF-α (Sigma-Aldrich, St. Louis, MO, USA; Cat. No. H8916), expressed in HEK293 cells and certified by the manufacturer as endotoxin-tested (≤1.00 EU/µg TNF-α, determined by LAL assay), was used for endothelial cell stimulation or human α-thrombin (Sigma-Aldrich, St. Louis, MO, USA) at the concentrations and incubation times specified for each experiment. After stimulation, we collected cells and culture supernatants for gene expression analysis, protein detection, cytokine quantification, TF expression, and procoagulant activity assessment.

### 2.6. RNA Isolation

We purified total RNA from HBEC-5i cells using the RNeasy Mini Kit (QIAGEN, Hilden, Germany) according to the manufacturer’s instructions. To remove residual genomic DNA, we performed an on-column DNase digestion using the RNase-Free DNase Set (QIAGEN; Cat. No. 79254). Briefly, after RNA bound to the RNeasy membrane and was washed with Buffer RW1, we treated the membrane-bound RNA with DNase I in Buffer RDD for 15 min at room temperature (20–30 °C). We then removed the DNase by additional washing steps and eluted the purified RNA in RNase-free water. We confirmed the absence of detectable genomic DNA contamination using No-RT controls during qPCR analysis, which showed no amplification. We measured RNA purity and concentration at 260 and 280 nm using a NanoDrop spectrophotometer (Thermo Fisher Scientific, Waltham, MA, USA). We considered samples with an A260/A280 ratio between 1.8 and 2.0 suitable for downstream applications. We stored purified RNA at −80 °C until complementary DNA (cDNA) synthesis. We verified RNA integrity before cDNA synthesis.

### 2.7. Reverse Transcription and Quantitative Real-Time PCR

First-strand cDNA was synthesized from 250 ng of total RNA using the iScript^TM^ cDNA Synthesis Kit (Bio-Rad Laboratories, Hercules, CA, USA) according to the manufacturer’s instructions. Quantitative real-time PCR (RT-qPCR) was performed using SsoFast^TM^ EvaGreen^®^ Supermix (Bio-Rad Laboratories) on a CFX96 Touch^TM^ real-time PCR detection system (Bio-Rad Laboratories). We performed all reactions in triplicate using the manufacturer-recommended final volume and included no-template controls to rule out reagent contamination. Table 1 shows the templates used and the resulting amplification products. We normalized gene expression levels to the housekeeping gene glyceraldehyde-3-phosphate dehydrogenase (GAPDH), which served as the endogenous reference across all experiments. We calculated relative transcript abundance using the comparative 2^−ΔΔCt^ method, with untreated HBEC-5i cells collected at the initial experimental time point serving as the calibrator. Relative expression values were reported as fold change relative to the corresponding control group.

### 2.8. Western Blot Analysis

Whole-cell protein extracts were prepared using RIPA lysis buffer supplemented with protease and phosphatase inhibitor cocktails (Thermo Fisher Scientific). Protein concentrations were measured using the Bio-Rad Protein Assay (Bradford method; Bio-Rad Laboratories) according to the manufacturer’s instructions.

Equal amounts of protein (10 μg) were separated by SDS-PAGE on 12% polyacrylamide gels and transferred to polyvinylidene fluoride (PVDF) membranes (Millipore, Burlington, MA, USA). Membranes were blocked with 5% non-fat dry milk in Tris-buffered saline containing 0.1% Tween-20 (TBST) for 1 h at room temperature. For Western blot analysis. Membranes were incubated overnight at 4 °C with the following primary antibodies: mouse monoclonal anti-Tissue Factor antibody (TF; clone H-9, Santa Cruz Biotechnology, Dallas, TX, USA; Cat. No. sc-374441; IgG2bκ; dilution 1:500), mouse monoclonal anti-vinculin antibody (clone 7F9, Santa Cruz Biotechnology; Cat. No. sc-73614; IgG1κ; dilution 1:800), used as the loading control, and mouse monoclonal anti-Influenza A virus Matrix Protein 1 antibody (M1; clone GA2B, Abcam, Cambridge, UK; Cat. No. ab22396; IgG1; dilution 1:100), which specifically recognizes Influenza A virus M1 and was used to assess viral protein expression in infected HBEC-5i cells. After incubation with the primary antibodies, we washed the membranes and incubated them with the corresponding HRP-conjugated secondary antibody. We visualized immunoreactive proteins using an enhanced chemiluminescence detection system.

After washing with TBST, we incubated membranes with the appropriate horseradish peroxidase-conjugated secondary antibodies for 1 h at room temperature. We visualized immunoreactive bands using Clarity Western ECL substrate (Bio-Rad Laboratories) and detected them with a ChemiDoc Imaging System (Bio-Rad Laboratories). We quantified band intensities using Image Lab software v5.0 (Bio-Rad Laboratories) and normalized them to vinculin expression.

### 2.9. Enzyme-Linked Immunosorbent Assay (ELISA)

We quantified TNF-α secretion in cell culture supernatants using commercially available human TNF-α ELISA kits (BioLegend, San Diego, CA, USA) per the manufacturer’s instructions. We collected supernatants at the indicated time point after influenza infection or stimulation, centrifuged them to remove cellular debris, and stored them at −80 °C until analysis. Absorbance was measured at 450 nm using a microplate reader (Agilent BioTek, Winooski, VT, USA), and cytokine concentrations were calculated from a standard curve generated with the kit’s recombinant human TNF-α. We analyzed all samples and standards in duplicate and reported TNF-α concentrations in pg/mL.

### 2.10. Tissue Factor Activity Assay

We determined TF procoagulant activity using the Tissue Factor Activity Assay Kit (Human, Colorimetric; Abcam, Cambridge, UK; ab108906) according to the manufacturer’s instructions. We lysed HBEC-5i cells under the indicated experimental conditions in 50 mM Tris-buffered saline (pH 8.0) containing 0.1% Tween-20 for 30 min on ice. We clarified cell lysates by centrifugation and used the resulting supernatants for the activity assay. The assay measures the functional ability of the TF/FVIIa complex to activate Factor X (FX) to Factor Xa (FXa). For each reaction, add 70 μL of assay mixture to each well, then add 10 μL of sample or recombinant human TF standard. Incubate plates for 30 min at 37 °C. Next, add 20 μL of FXa-specific chromogenic substrate and measure absorbance at 405 nm every 5 min for 30 min using a BioTek microplate reader.

For data analysis, we calculated the mean absorbance of replicate wells and used the linear portion of the reaction to determine the reaction rate as ΔOD405/min.

ΔOD405/min = (ODfinal − ODinitial)/reaction time (min).

We generated a standard curve using recombinant human TF concentrations of 0, 7.81, 15.63, 31.25, 62.5, 125, and 250 pM. We calculated TF activity in experimental samples by interpolating from the standard curve using the linear portion of the assay response and reported the results as pM TF equivalents.

Because the assay quantifies the ability of sample-associated TF/FVIIa activity to generate FXa from exogenous FX, the results were interpreted as a functional measure of TF-associated procoagulant activity.

### 2.11. Statistical Analysis

All experiments included at least three independent biological replicates. Data are presented as mean ± standard error of the mean (SEM). Statistical analyses were performed using GraphPad Prism version 11.0 (GraphPad Software, San Diego, CA, USA). We compared two groups using an unpaired, two-tailed Student’s *t*-test. We analyzed comparisons among groups using one- or two-way analysis of variance (ANOVA) with Dunnett’s multiple-comparisons test, as appropriate. We considered differences statistically significant at *p* < 0.05.

## 3. Results

### 3.1. Influenza A Virus (H1N1) pdm09 Infection Is Associated with Viral Gene and Protein Expression in HBEC-5i Cells

To determine whether HBEC-5i cells we evaluated viral M-gene RNA and M1 protein expression at multiple time points after exposure to Influenza A virus (H1N1) pdm09. RT-qPCR analysis detected M-gene RNA, with viral RNA levels increasing over time. Consistent with this, immunoblot analysis detected M1 protein and showed increased M1 abundance over the post-infection period evaluated. Together, these findings demonstrate Influenza A virus-associated gene and protein expression in HBEC-5i cells. However, because we did not directly assess infectious progeny production, these results should not be interpreted as definitive evidence of productive viral infection.

RT-qPCR revealed a progressive increase in viral matrix M gene transcripts throughout infection, indicating active viral gene and protein expression in HBEC-5i cells (Figure 1A). These findings suggest that brain endothelial cells may be permissive to Influenza A virus (H1N1) pdm09 infection rather than acting solely as passive targets of circulating inflammatory mediators, and they support the expression of intracellular viral infection-associated markers in infected endothelial cells.

We assessed viral protein synthesis by Western blot analysis. Consistent with the transcriptional data, Influenza A matrix protein M1 expression increased 24 h post-infection (Figure 1B,C), corroborating the intracellular viral infection-associated expression observed in infected endothelial cells.

Taken together, these results establish HBEC-5i cells as a suitable in vitro model for investigating the direct effects of the Influenza A virus (H1N1) pdm09 on the cerebral endothelium. More importantly, they show that viral infection of endothelial cells could initiate the inflammatory and procoagulant responses described in the following sections, providing an experimental basis for investigating the mechanisms underlying the development of an endothelial immunothrombosis phenotype.

### 3.2. Influenza A Virus (H1N1) pdm09 Triggers an Endothelial Pro-Inflammatory Response Characterized by TNF-α Production

Having established that HBEC-5i cells support productive Influenza A virus (H1N1) pdm09 infection, we next investigated whether viral replication triggered an inflammatory response in brain endothelial cells. Because endothelial activation is a critical step in initiating immunothrombosis, we first assessed the expression and secretion of the pro-inflammatory cytokine TNF-α.

RT-qPCR analysis showed significant upregulation of TNF-α mRNA in infected HBEC-5i cells compared with uninfected cells (Figure 2A). Consistent with these findings, ELISA detected a significant increase in TNF-α secretion in the culture supernatant after viral infection (Figure 2B), suggesting that infected endothelial cells may actively help establish a local pro-inflammatory microenvironment.

The temporal pattern of TNF-α induction closely mirrored the kinetics of viral replication, suggesting that endothelial inflammatory activation may be directly linked to productive Influenza A virus (H1N1) pdm09 infection rather than to secondary exposure to circulating inflammatory mediators. This coordinated increase in TNF-α gene expression and protein secretion demonstrates that Influenza virus infection induces an inflammatory state in cerebral endothelial cells. Together, these findings identify TNF-α as an early endothelial response to Influenza A virus (H1N1) pdm09 infection and provide a mechanistic basis for investigating whether this inflammatory activation promotes the acquisition of a procoagulant phenotype characteristic of endothelial immunothrombosis.

### 3.3. Influenza A Virus (H1N1) pdm09 Promotes Endothelial Tissue Factor Expression in Human Brain Endothelial Cells

Having shown that Influenza A virus (H1N1) pdm09 infection induces a strong TNF-α-mediated inflammatory response in HBEC-5i cells, we next investigated whether this inflammatory activation was accompanied by TF induction, the principal initiator of the extrinsic coagulation pathway and a key mediator of endothelial immunothrombosis.

RT-qPCR analysis revealed a significant increase in TF mRNA expression in infected endothelial cells compared with uninfected controls (Figure 3A). This transcriptional upregulation was accompanied by a marked increase in TF protein abundance, as determined by Western blotting (Figure 3C,D), confirming that Influenza A virus (H1N1) pdm09 infection induces TF expression at both the gene and protein levels.

Notably, TF induction followed the increase in TNF-α expression observed in the previous experiments, supporting the idea that inflammatory signaling contributes to endothelial procoagulant activation during viral infection. Although these experiments do not establish causality, the temporal association between TNF-α induction and TF upregulation suggests that inflammatory mediators contribute to the molecular events that lead to endothelial dysfunction. Together, these findings suggest that Influenza A virus (H1N1) pdm09 infection extends beyond inducing a pro-inflammatory response by initiating a coordinated procoagulant program in brain endothelial cells. TF upregulation is an early molecular event linking endothelial inflammation to activation of the extrinsic coagulation pathway and provides a basis for evaluating whether this newly expressed TF is functionally active.

### 3.4. Influenza A Virus (H1N1) pdm09 Confers Functional Procoagulant Activity to Brain Endothelial Cells

Having established that Influenza A virus (H1N1) pdm09 infection induces TF expression, we next determined whether this molecular response translated into functional activation of the extrinsic coagulation pathway. To address this, we assessed TF-dependent procoagulant activity by measuring activated FXa generation.

Influenza A virus (H1N1) pdm09-infected HBEC-5i cells exhibited a significant increase in TF-dependent FXa generation compared with uninfected controls (Figure 3B). Notably, TF-associated procoagulant activity increased as early as 0.5 h after viral exposure, preceding the major changes in TF gene and protein expression. This rapid response is consistent with early functional activation or increased availability of pre-existing endothelial TF rather than de novo TF synthesis, demonstrating that the newly expressed TF was functionally active. These findings provide functional evidence that Influenza A virus (H1N1) pdm09 infection activates the extrinsic coagulation pathway in brain endothelial cells. Notably, the acquisition of TF-dependent procoagulant activity indicates that influenza-induced endothelial activation extends beyond transcriptional and protein-level changes, resulting in a biologically relevant procoagulant phenotype.

### 3.5. TNF-α Directly Drives Endothelial Tissue Factor Expression and Procoagulant Activity

Having showed that Influenza A virus (H1N1) pdm09 infection induces TNF-α production and TF-dependent procoagulant activity; we investigated whether TNF-α directly contributes to endothelial procoagulant activation. To address this, we stimulated HBEC-5i cells with recombinant human TNF-α and evaluated TF expression and functional activity. We observed that TNF-α stimulation significantly increased TF mRNA expression and protein abundance compared with unstimulated cells (Figure 4A,C,D), confirming that TNF-α directly promotes TF expression in brain endothelial cells.

To determine whether increased TF expression translated into functional procoagulant activity, we measured TF-dependent FXa generation following TNF-α stimulation. TNF-α significantly increased TF activity relative to control cells (Figure 4B), demonstrating that cytokine-induced TF is functionally active and capable of initiating the extrinsic coagulation pathway.

### 3.6. Thrombin Amplifies Endothelial Inflammatory Activation

Next, we investigated whether thrombin—the primary distal effector of the coagulation cascade—amplifies inflammatory activation in endothelial cells. We stimulated HBEC-5i cells with recombinant human thrombin and quantified TNF-α expression and secretion.

Stimulation of endothelial cells with recombinant human thrombin significantly increased TNF-α mRNA expression compared with untreated cells (Figure 5A). This transcriptional response was accompanied by increased TNF-α secretion (Figure 5B), suggesting that thrombin could directly activate inflammatory signaling in cerebral endothelial cells. Thrombin’s ability to stimulate TNF-α production indicates that coagulation cascade activation actively reinforces endothelial inflammation rather than merely serving as a terminal hemostatic event. These findings support bidirectional communication between inflammatory and coagulation pathways during Influenza A virus (H1N1) pdm09 infection.

Taken together, these results suggest that thrombin may amplify endothelial inflammatory activation, establishing a positive feedback loop in which coagulation signaling sustains cytokine production. This reciprocal interaction provides the mechanistic basis for the persistent inflammatory response that precedes the development of an immunothrombotic endothelial phenotype.

### 3.7. Thrombin Reinforces the Endothelial Procoagulant Phenotype

Having demonstrated that thrombin amplifies endothelial inflammatory activation, we next investigated whether this enzyme also enhances the endothelial procoagulant response by regulating tissue factor (TF) expression and activity. Stimulation of HBEC-5i cells with recombinant human thrombin significantly increased TF mRNA expression compared with untreated cells (Figure 6A). This transcriptional response was accompanied by increased TF protein abundance (Figure 6C,D), indicating that thrombin promotes TF expression in cerebral endothelial cells.

To determine whether thrombin-induced TF was functionally active, we measured thrombin-stimulated TF-dependent FXa generation. Thrombin stimulation significantly increased TF procoagulant activity compared with control cells (Figure 6B), demonstrating that thrombin not only enhances TF expression but also promotes its functional activation.

## 4. Discussion

This study identifies a mechanism by which Influenza A virus (H1N1) pdm09 infection could reprogram human cerebral microvascular endothelial cells toward an immunothrombotic endothelial phenotype. Our findings suggest a mechanistic model in which productive viral infection activates a TNF-α–TF–thrombin positive-feedback signaling loop that functionally couples inflammation and coagulation pathways, sustaining endothelial activation beyond the initial viral insult. By demonstrating that thrombin amplifies both inflammatory signaling and tissue factor (TF)-dependent procoagulant activity, this study provides new mechanistic insights into how vascular dysfunction may contribute to the pathogenesis of severe influenza.

Although Influenza A virus (IAV) primarily infects respiratory epithelial cells, evidence indicates that endothelial dysfunction is a key determinant of disease severity. Endothelial activation increases vascular permeability and leukocyte recruitment, amplifies cytokine responses, and triggers the coagulation cascade, ultimately promoting diffuse alveolar damage, acute respiratory distress syndrome, and multiple organ failure [3,4]. Histopathological studies of fatal influenza cases have consistently shown endothelial activation, fibrin deposition, microvascular thrombosis, and widespread vascular inflammation, supporting the view that the endothelium actively participates in disease progression rather than merely serving as a passive target of systemic inflammation [19,20,21,22]. In this context, our findings expand the current understanding of influenza-associated vascular pathology by demonstrating that human cerebral microvascular endothelial cells are susceptible to productive infection by the Influenza A virus (H1N1) pdm09virus and undergo coordinated inflammatory and procoagulant activation.

The susceptibility of brain endothelial cells to productive infection has important implications for the neurological manifestations frequently observed in severe influenza, including encephalopathy, cerebrovascular complications, and blood–brain barrier dysfunction [22,23,24]. Although previous studies have largely focused on pulmonary endothelial responses, our results suggest that cerebral endothelial activation may also contribute to influenza-associated neurovascular pathology via inflammatory and coagulation mechanisms.

A central finding of this study is a direct molecular link between inflammatory signaling and endothelial coagulation activation. TNF-α is a well-established mediator of influenza-associated hyperinflammation and has been implicated in increased vascular permeability, leukocyte adhesion, oxidative stress, and endothelial dysfunction [20]. Our data demonstrate that influenza-induced TNF-α production precedes TF upregulation and that recombinant TNF-α recapitulates the induction of TF expression and activity observed after viral infection. These observations support TNF-α as a major upstream regulator linking influenza-induced inflammation to endothelial procoagulant activation, consistent with its known ability to induce TF transcription via the NF-κB and AP-1 pathways [7,25,26,27].

Beyond establishing this inflammatory-to-coagulation axis, our study further shows that thrombin functions as an active signaling molecule rather than merely the terminal protease of the coagulation cascade. Consistent with previous reports, thrombin activates PAR-dependent signaling pathways that regulate cytokine production, endothelial permeability, leukocyte recruitment, and vascular remodeling [28,29,30]. In this study, thrombin significantly increased both TNF-α production and TF-dependent procoagulant activity in HBEC-5i cells, indicating that coagulation activation actively reinforces endothelial inflammation.

We demonstrated that inflammatory and coagulation pathways are not activated independently but instead integrate into a self-reinforcing signaling network. Rather than being parallel consequences of viral infection, TNF-α, TF, and thrombin participate in a feed-forward signaling circuit in which inflammatory activation promotes coagulation, and coagulation signaling further amplifies inflammatory and procoagulant responses. This reciprocal amplification provides a plausible mechanistic explanation for the persistence of endothelial dysfunction and microvascular thrombosis observed during severe influenza, even after the initial antiviral response has been established [27].

These findings could reinforce the broader concept of immunothrombosis, in which innate immune responses and coagulation mechanisms cooperate to contain pathogen dissemination but, when dysregulated, contribute to endothelial injury, tissue ischemia, microvascular thrombosis, and organ dysfunction [3,11]. Although immunothrombosis has been extensively studied in severe COVID-19 cases [31,32], the molecular mechanisms underlying endothelial immunothrombosis during influenza infection remain less characterized. Our results suggest that Influenza A virus (H1N1) pdm09 can activate inflammatory and procoagulant endothelial signaling pathways that share key mechanistic features with those described in severe SARS-CoV-2 infection, supporting the idea that endothelial immunothrombosis may be a conserved pathogenic mechanism across respiratory viral infections (Figure 7 [30,33]).

Beyond advancing mechanistic understanding of influenza pathogenesis, these findings may also have therapeutic implications. Identifying the TNF-α–TF–thrombin signaling axis highlights several potential therapeutic targets, including modulating inflammatory cytokines, inhibiting TF activity, blocking thrombin, or interfering with PAR-mediated signaling. Although these strategies require validation in appropriate preclinical models, targeting endothelial immunothrombotic signaling may be a promising adjunct to antiviral therapy to limit vascular injury, inflammation, and thrombotic complications during severe influenza.

Several limitations warrant acknowledgment. First, an immortalized human brain microvascular endothelial cell line does not fully recapitulate the complexity of the neurovascular microenvironment. Second, although our findings strongly implicate TNF-α in TF induction, further studies using neutralizing antibodies or pharmacological inhibition of NF-κB, PAR-1, or TF signaling are needed to establish a direct causal relationship. Third, we did not assess the contributions of other inflammatory mediators or endogenous anticoagulant pathways that may also modulate endothelial dysfunction during influenza infection. Despite these limitations, our findings provide evidence that influenza virus infection promotes a coordinated proinflammatory and procoagulant response in human brain endothelial cells by activating the TNF-α–TF axis. These results identify a mechanistic link among viral infection, endothelial inflammation, and coagulation activation, thereby extending current knowledge of mechanisms that may contribute to influenza-associated neurovascular complications.

Another limitation of the present study is that we did not directly demonstrate productive Influenza A virus (H1N1) pdm09 infection by quantifying infectious progeny. Although time-dependent detection of viral M-gene RNA and M1 protein provides evidence of infection-associated molecular activity in HBEC-5i cells, a plaque assay, TCID50, or equivalent infectivity-based analyses of progeny virus would be needed to conclusively establish productive infection.

A limitation of this study is the lack of quantitative assessment of cell viability at 24 and 48 h post-infection. Although routine microscopy did not reveal a clear loss of the endothelial monolayer, infection-related cytotoxicity could have influenced responses at later stages. Future research should include quantitative viability and cytotoxicity assays to better distinguish endothelial activation from cellular damage caused by prolonged influenza A virus infection. Although our findings support a functional association between influenza-induced inflammatory activation and endothelial procoagulant responses, they do not establish a causal TNF-α–TF–thrombin feed-forward circuit. Recombinant TNF-α can induce TF, supporting TNF-α as a potential upstream mediator of endothelial TF expression. Still, it does not demonstrate that endogenous TNF-α is required for the TF response elicited by influenza infection. Likewise, the observed activation of TF-dependent coagulation is consistent with downstream thrombin generation. Still, it does not establish that thrombin/PAR-1 signaling subsequently amplifies TNF-α or TF expression in this experimental system. Therefore, the TNF-α–TF–thrombin/PAR-1 axis presented here should be regarded as a proposed mechanistic model integrating our experimental observations with established inflammatory and coagulation signaling pathways. Future loss-of-function studies using TNF-α/TNFR neutralization, TF silencing or functional blockade, and pharmacological or genetic inhibition of thrombin/PAR-1 signaling will be required to establish causality and determine whether these pathways constitute a self-amplifying endothelial immunothrombotic circuit during influenza A virus infection.

## 5. Conclusions

In conclusion, our findings demonstrate that influenza A(H1N1) pdm09 infection promotes inflammatory and procoagulant activation in human brain microvascular endothelial cells. The induction of TNF-α and TF, along with recombinant TNF-α’s ability to reproduce TF upregulation and increase TF-dependent procoagulant activity, supports a functional association between endothelial inflammation and coagulation. These observations are consistent with a potential TNF-α–TF–thrombin/PAR-1 amplification mechanism that may contribute to endothelial immunothrombosis during severe influenza. However, targeted loss-of-function approaches are needed to establish causal relationships among these components. Thus, the proposed pathway should be considered a mechanistic framework for future investigation rather than a fully demonstrated feed-forward circuit.

## Figures and Tables

**Figure 1:**
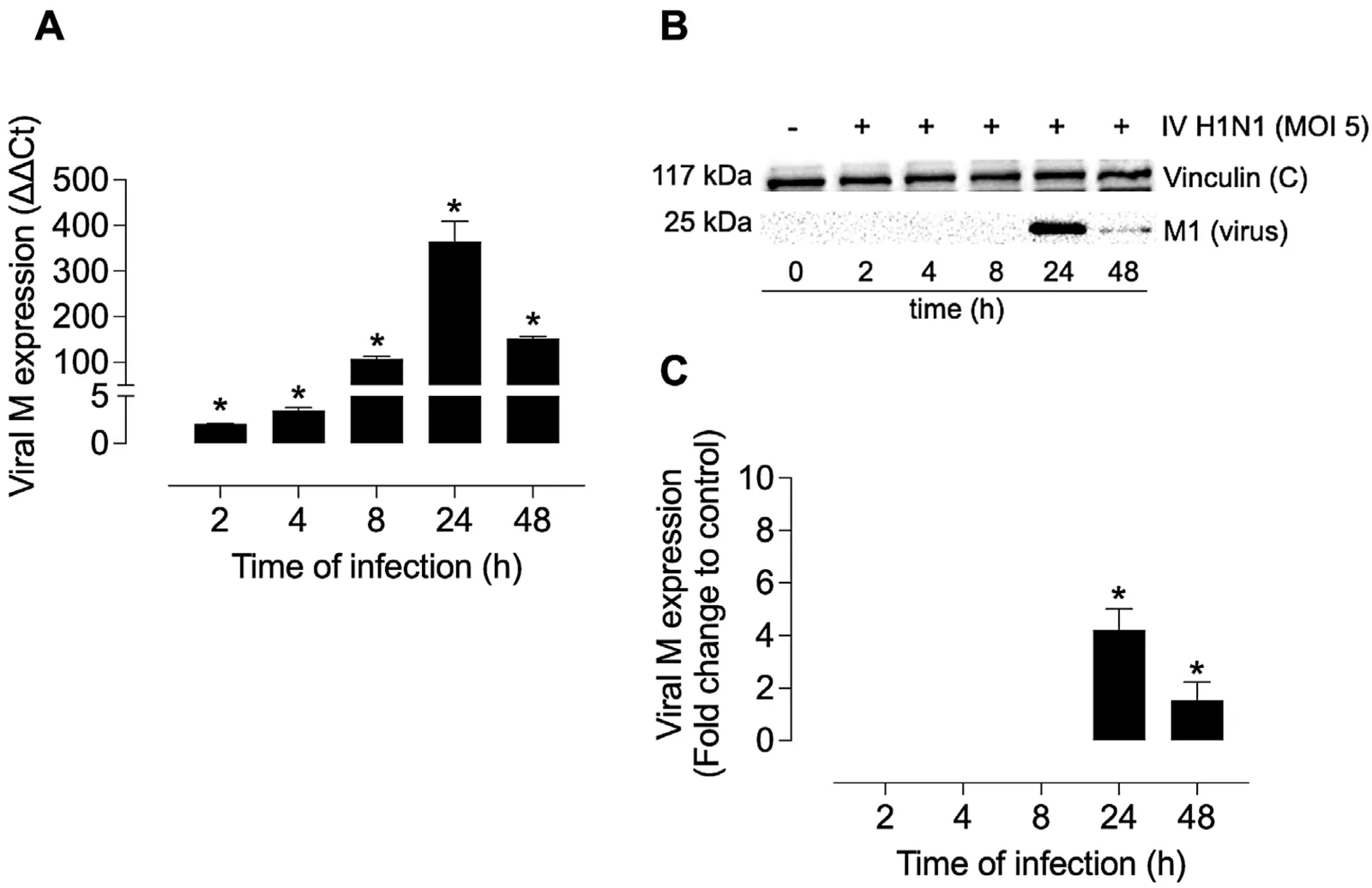
Influenza A virus (H1N1) pdm09 infection in HBEC-5i cells. (**A**) Viral M1 mRNA expression was assessed by RT-qPCR in HBEC-5i cells infected with Influenza A/H1N1 (MOI = 5) at the indicated time points. (**B**) Representative Western blot showing viral M1 protein expression after infection. Vinculin served as the loading control. (**C**) Densitometric analysis of M1 protein expression, normalized to vinculin and expressed as a fold change relative to uninfected cells. Data are presented as the mean ± SEM of three independent experiments. * Shows significant differences compared to uninfected cells (*p* < 0.05).

**Figure 2:**
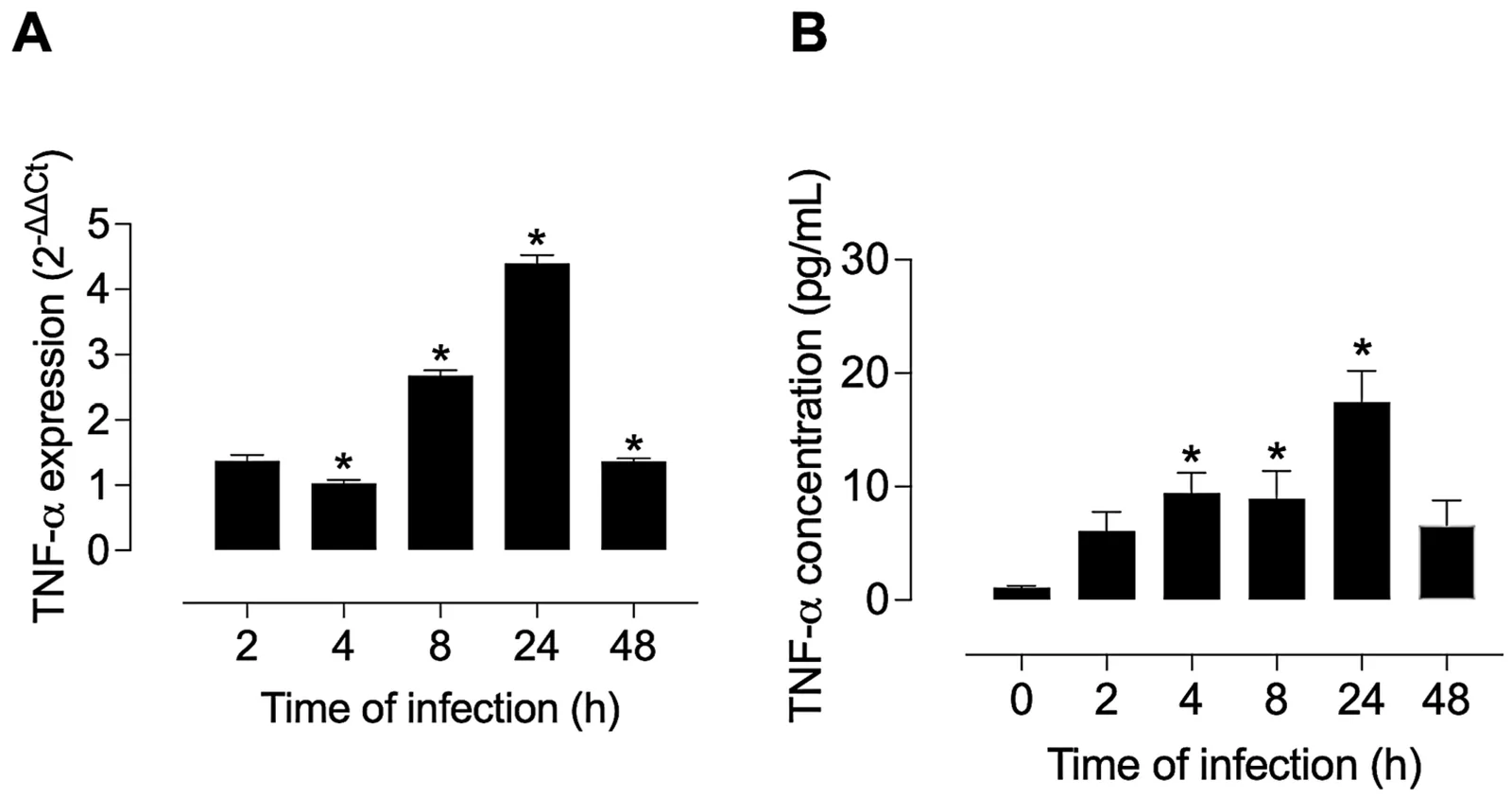
Influenza A virus (H1N1) pdm09 induces TNF-α expression and secretion in HBEC-5i. (**A**) RT-qPCR assessed TNF-α mRNA expression in HBEC-5i infected with Influenza A/H1N1 (MOI = 5) at the indicated time points. (**B**) ELISA quantified TNF-α protein levels in culture supernatants. Data are presented as the mean ± SEM from three independent experiments. * Shows significant differences compared to uninfected cells (*p* < 0.05).

**Figure 3:**
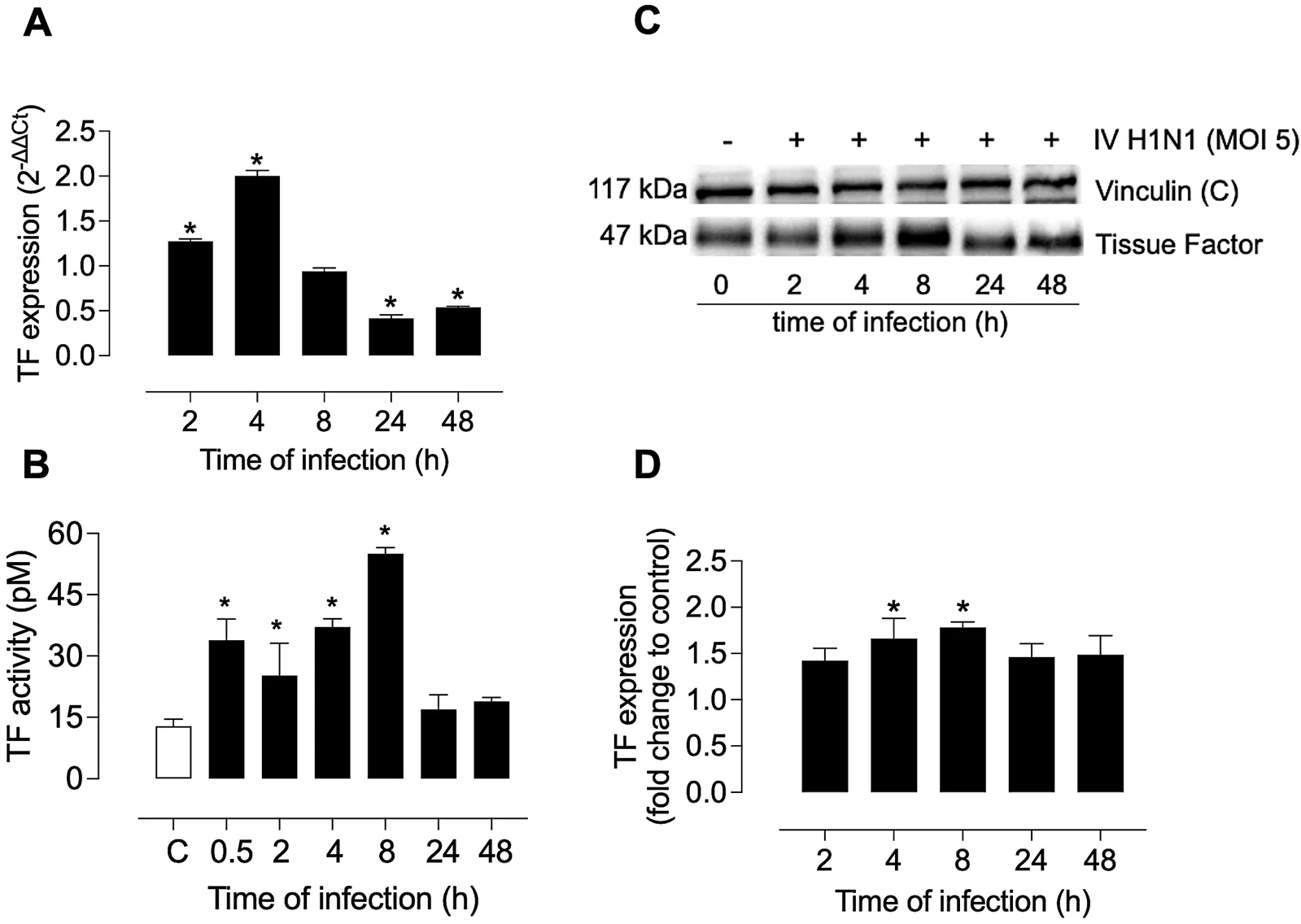
Influenza A Virus (H1N1) pdm09 induces TF expression and procoagulant activity in HBEC-5i. (**A**) TF mRNA expression was analyzed by RT-qPCR in HBECs infected with Influenza A/H1N1 (MOI = 5) at the indicated time points. (**B**) TF procoagulant activity was measured by FXa generation. (**C**) Representative Western blot showing TF protein expression after infection. Vinculin served as the loading control. (**D**) Densitometric analysis of TF protein expression, normalized to vinculin and expressed as a fold change relative to uninfected cells. Data are presented as the mean ± SEM of three independent experiments. * Shows significant differences compared to uninfected cells (*p* < 0.05).

**Figure 4:**
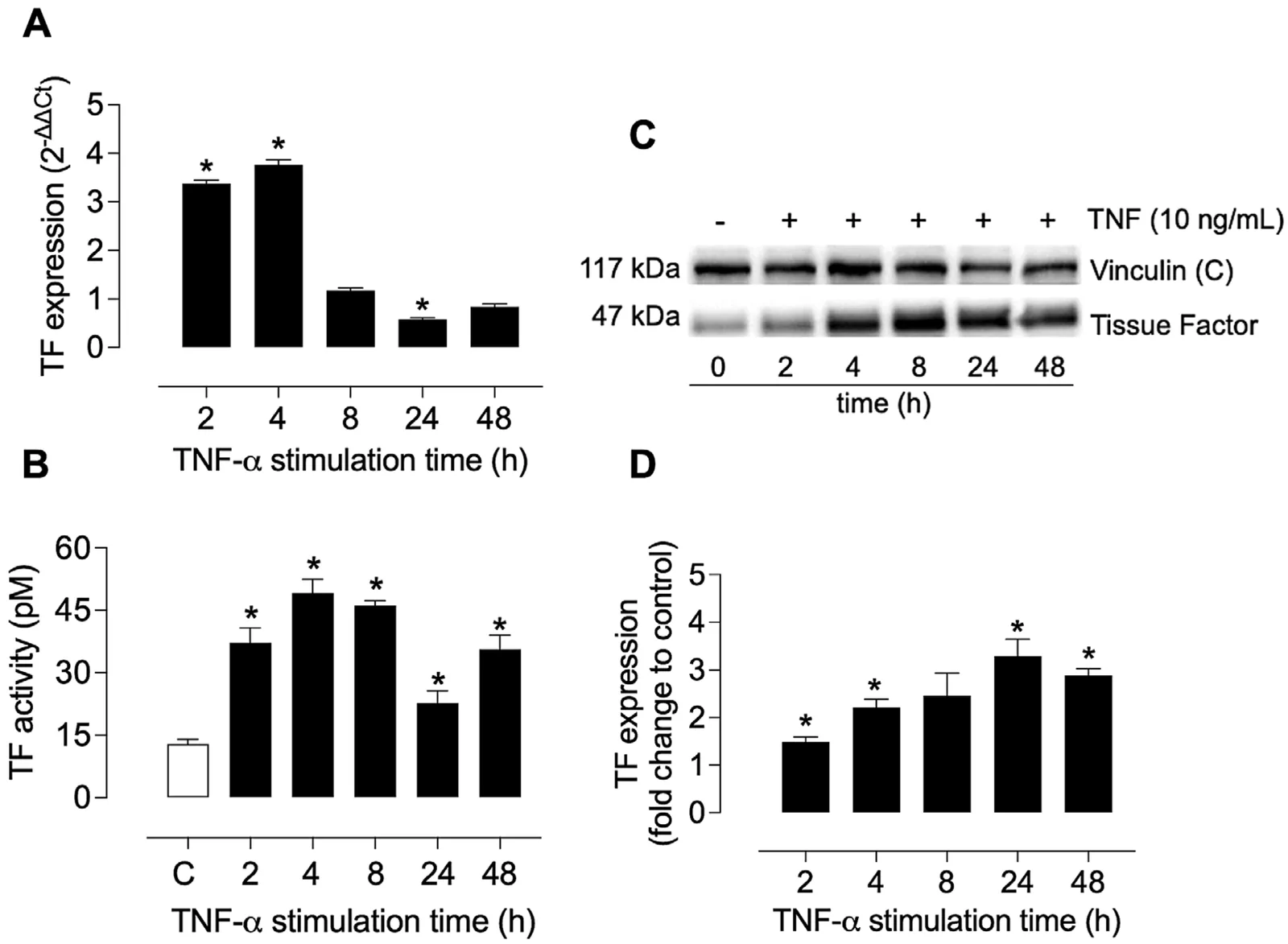
TNF-α induces TF expression and procoagulant activity in HBEC-5i. (**A**) TF mRNA expression was assessed by RT-qPCR in HBECs stimulated with TNF-α (10 ng/mL) at the indicated time points. (**B**) TF procoagulant activity was quantified by measuring FXa generation. (**C**) Representative Western blot showing TF protein expression after TNF-α stimulation. Vinculin served as the loading control. (**D**) Densitometric analysis of TF protein expression, normalized to vinculin and expressed as a fold change relative to unstimulated cells. Data are presented as the mean ± SEM of three independent experiments. * Shows significant differences compared to uninfected cells (*p* < 0.05).

**Figure 5:**
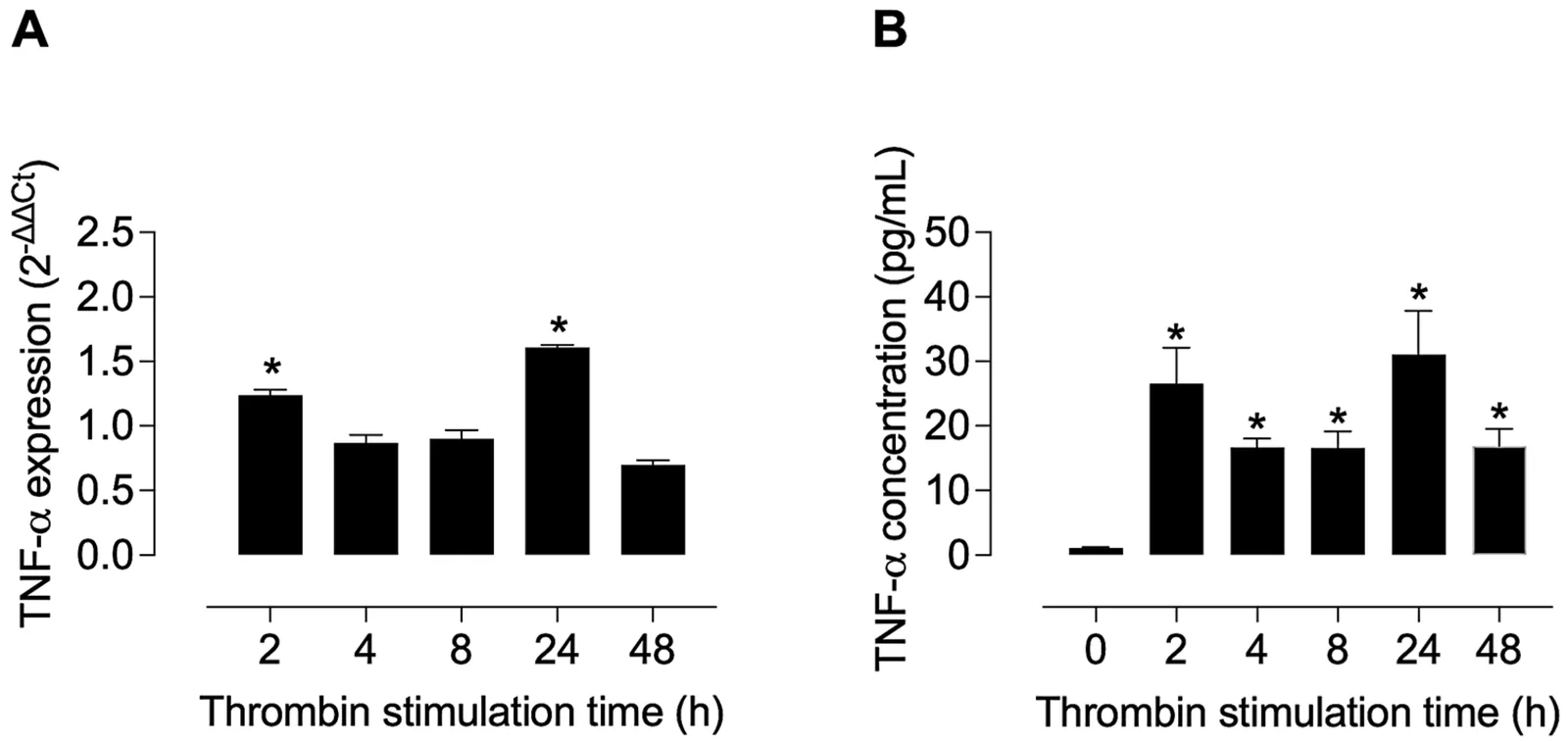
Thrombin induces TNF-α expression and secretion in HBEC-5i. (**A**) TNF-α mRNA expression was assessed by RT-qPCR in HBECs stimulated with thrombin (5 U/mL) at the indicated time points. (**B**) TNF-α protein concentration in culture supernatants was quantified by ELISA. Data are presented as the mean ± SEM from three independent experiments. * Shows significant differences compared to uninfected cells.

**Figure 6:**
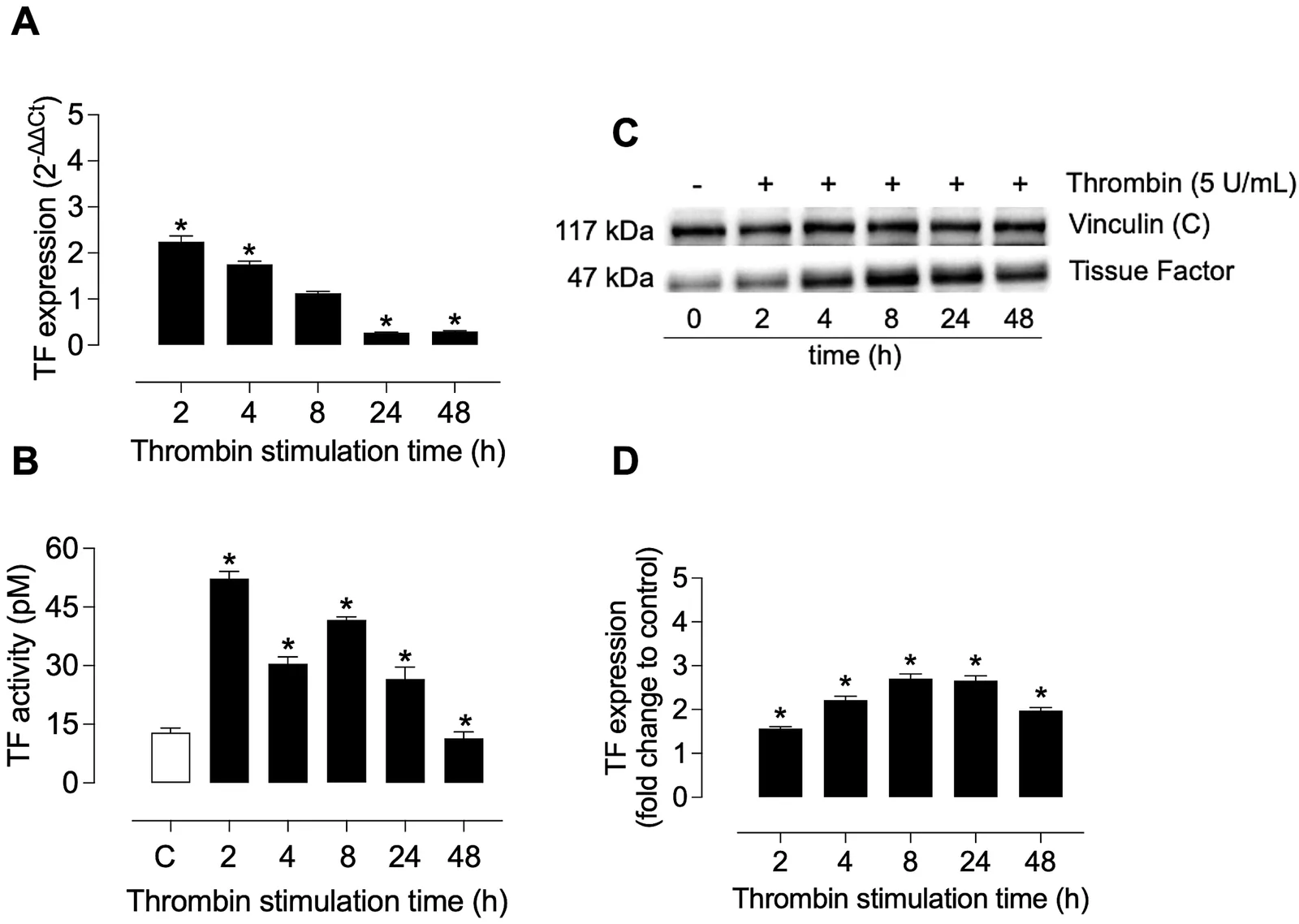
Thrombin induces TF expression and procoagulant activity in HBEC-5i. (**A**) TF mRNA expression was analyzed by RT-qPCR in HBECs stimulated with thrombin (5 U/mL) at the indicated time points. (**B**) TF procoagulant activity was assessed by measuring FXa generation. (**C**) Representative Western blot showing TF protein expression after thrombin stimulation. Vinculin served as the loading control. (**D**) Densitometric analysis of TF protein expression, normalized to vinculin and expressed as a fold change relative to unstimulated cells. Data are presented as the mean ± SEM from three independent experiments. * Shows significant differences compared to uninfected cells.

**Figure 7:**
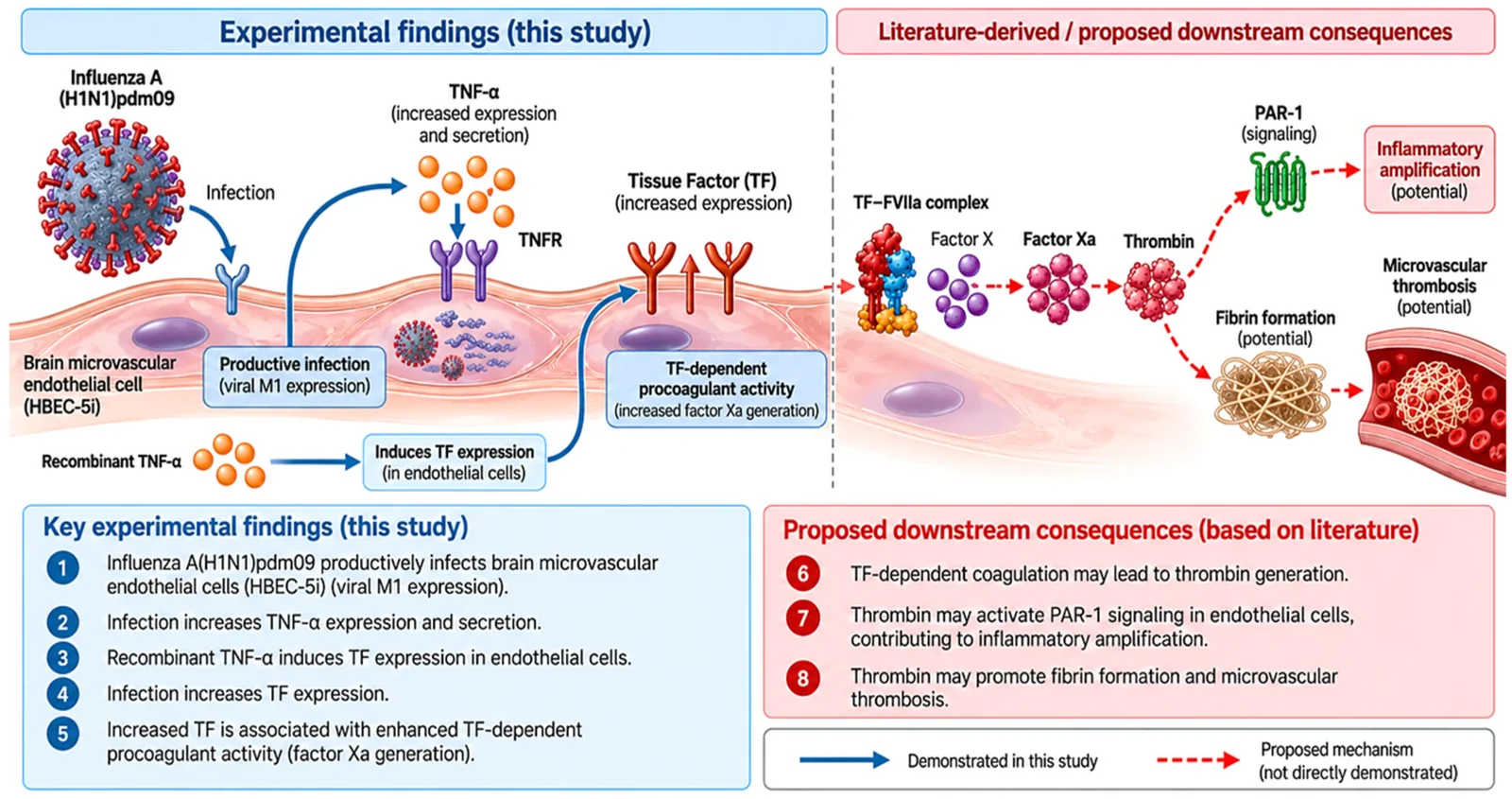
Experimental findings and proposed downstream consequences of influenza-induced endothelial procoagulant activation. Influenza A virus (H1N1) pdm09 productively infects microvascular endothelial cells (HBEC-5i), leading to increased TNF-α expression and secretion. Recombinant TNF-α induces TF expression, and infection increases TF expression and TF-dependent procoagulant activity, as evidenced by enhanced factor Xa generation. Based on established coagulation biology, the subsequent steps, including thrombin generation, PAR-1 signaling, fibrin formation, and microvascular thrombosis, are proposed as potential downstream consequences and not directly examined in this study.

**Table 1:** Primers used for quantitative RT-PCR.

Gene	Sequence	Fragment Size	References
Tissue factor	Fw: gcc cgg tag agt gta tgg gcc ag	145 bp	[15]
	Rv: gct ctg ccc cac tcc tgc ctt		
Viral gene M	Fw: aag acc aat cct gtc acc tct ga	95 bp	[16]
	Rv: caa agc gtc tac gct gag tcc		
GAPDH	Fw: gga ttt ggt cgt att ggg	204 bp	[17]
	Rv: gga aga tgg tga tgg gat t		
TNF-α	Fw: agc cca tgt tgt agc aaa cc	133 bp	[18]
	Rv: tga ggt aca ggc cct ctg at		

## Data Availability

The original contributions presented in this study are included in the article; please direct further inquiries to the corresponding author.

## Abbreviations

AP-1	Activator Protein-1
ATCC	American Type Culture Collection
ANOVA	Analysis of Variance
cDNA	complementary DNA
COVID-19	Coronavirus Disease 2019
DMEM/F12	Dulbecco’s Modified Eagle Medium/F-12
ECGS	Endothelial Cell Growth Supplement
EMEM	Eagle’s Minimum Essential Medium
ELISA	Enzyme-Linked Immunosorbent Assay
FX	factor X
FVII	factor VII
FBS	Fetal Bovine Serum
GAPDH	Glyceraldehyde-3-Phosphate Dehydrogenase
HBEC-5i	Human Brain Endothelial Cells
IAV	Influenza A virus
MDCK	Madin-Darby canine kidney
MOI	Multiplicity of Infection
NF-κB	Nuclear factor kappa-light-chain-enhancer of activated B cells
PAR	Protease-Activated Receptors
PBS	Phosphate-Buffered Saline
PFU	Plaque-Forming Units
PVDF	Polyvinylidene Fluoride
RIPA	Radioimmunoprecipitation assay buffer
RT-qPCR	Quantitative Reverse Transcription Polymerase Chain Reaction
SEM	Standard Error of the Mean
SDS-PAGE	Sodium Dodecyl Sulfate-Polyacrylamide Gel Electrophoresis
SARS-CoV-2	Severe Acute Respiratory Syndrome Coronavirus 2
TF	Tissue Factor
TPCK	L-1-tosylamido-2-phenyletyl chloromethyl ketone
TBST	Tris-buffered saline Tween-20
TNF-α	Tumor necrosis factor-alpha
